# Apelin enhances IL-1β expression in human synovial fibroblasts by inhibiting miR-144-3p through the PI3K and ERK pathways

**DOI:** 10.18632/aging.103195

**Published:** 2020-05-18

**Authors:** Ting-Kuo Chang, Yu-Han Wang, Shu-Jui Kuo, Shih-Wei Wang, Chun-Hao Tsai, Yi-Chin Fong, Nan-Lin Wu, Shan-Chi Liu, Chih-Hsin Tang

**Affiliations:** 1 Department of Medicine, Mackay Medical College, New Taipei, Taiwan; 2 Division of Spine Surgery, Department of Orthopedic Surgery, MacKay Memorial Hospital, New Taipei, Taiwan; 3 Graduate Institute of Biomedical Science, China Medical University, Taichung, Taiwan; 4 School of Medicine, China Medical University, Taichung, Taiwan; 5 Department of Orthopedic Surgery, China Medical University Hospital, Taichung, Taiwan; 6 Graduate Institute of Natural Products, College of Pharmacy, Kaohsiung Medical University, Kaohsiung, Taiwan; 7 Department of Sports Medicine, College of Health Care, China Medical University, Taichung, Taiwan; 8 Department of Orthopaedic Surgery, China Medical University Beigang Hospital, Yunlin, Taiwan; 9 Department of Dermatology, MacKay Memorial Hospital, Taipei, Taiwan; 10 Department of Medical Education and Research, China Medical University Beigang Hospital, Yunlin, Taiwan; 11 Chinese Medicine Research Center, China Medical University, Taichung, Taiwan; 12 Department of Biotechnology, College of Health Science, Asia University, Taichung, Taiwan

**Keywords:** osteoarthritis, apelin, IL-1β, miRNA-144-3p

## Abstract

Much data suggests intersecting activities between the adipokine apelin (APLN) and the pathologic processes of obesity and osteoarthritis (OA), with APLN modulating cartilage, synovium, bone, and various immune cell activities. The synovium plays an important role in the pathogenesis of OA. We investigated the crosstalk between APLN, a major OA-related adipokine, and interleukin 1 beta (IL-1β), a major proinflammatory cytokine, in human OA synovial fibroblasts (OASFs). We showed that APLN stimulated the synthesis of IL-1β in a concentration- and time-dependent manner, which was mitigated by blockade of the PI3K and ERK pathway. We also showed that APLN inhibited the expression of miRNA-144-3p, which blocks IL-1β transcription; this suppression activity was reversed via blockade of the PI3K and ERK pathway. Moreover, pathologic changes in OA cartilage were rescued when APLN was silenced by shAPLN transfection both *in vitro* and *in vivo*. Our evidence is the first to show that APLN stimulates the expression of IL-1β by activating the PI3K and ERK pathway and suppressing downstream expression of miRNA-144-3p in OASFs. We also demonstrate that knockdown of APLN expression by shAPLN transfection ameliorated changes in OA cartilage severity. These results shed light on OA pathogenesis and suggest a novel treatment pathway.

## INTRODUCTION

Osteoarthritis (OA) is a multifactorial disease that manifests with synovial inflammation, cartilage destruction, joint swelling and pain [1, 2]. Among the various risk factors, obesity has been linked to the risk of developing OA [3, 4], with the Framingham Heart Study demonstrating a 1.5- to 2-fold higher risk of developing knee OA among people who are obese compared with slimmer individuals [5]. In a US population-based study investigating community-dwelling older individuals (aged ≥70 years), a 5 kg/m^2^ increase in BMI increased the likelihood of developing knee OA by 32% [6]. However, despite the observations suggesting a correlation between obesity and OA, the detailed mechanisms underlying this correlation are far from clear. What is known is that adipokines, multifunctional molecules secreted by adipose tissue, act as an intersecting link between obesity and OA by modulating the activities of cartilage, synovium, bone, and various immune cells [3, 7].

The synovium plays an important role in the pathogenesis of OA. The synthesis of chondrolytic enzymes and proinflammatory mediators by the inflamed synovium leads to cartilage destruction, which enhances synovial inflammation, forming a vicious cycle [8, 9]. OA synovial cells sustain arthritic pathology by excreting chondrolytic enzymes and inflammatory mediators [8, 10, 11]. Synovium-targeted therapy could theoretically slow OA progression and lessen the severity of OA symptoms [12, 13].

Apelin (APLN) is one adipokine that is established as a pivotal player in OA pathogenesis [14]. Early *in vitro* investigations suggested that APLN stimulates chondrocyte proliferation and significantly increases transcript levels of the catabolic factors matrix metalloproteinase (MMP)-1, -3 and -9, as well as the expression of the proinflammatory cytokine interleukin 1 beta (IL-1β) [14]. IL-1β is a major chondrolytic enzyme that induces the degradation of proteoglycan from cartilage and suppresses new proteoglycan synthesis [15–17].

Non-coding, single-stranded micro-ribonucleic acids (miRNAs) mediate the expression of target genes at the post-transcriptional level [18, 19]. 3'-untranslated region (3'-UTR) miRNAs base-pair with the seed sequence of target mRNA molecules and effectively suppress target gene expression [1, 20]. While both APLN and IL-1β are known to be involved in the pathogenesis of OA, no details exist as to any interaction between these molecules in OA synovial cells. In view of the importance of synovial cells in OA pathogenesis, we explored the crosstalk between APLN and IL-1β in human osteoarthritis synovial fibroblasts (OASFs). Myriads of miRNAs are involved in OA pathogenesis [1, 8]. We hypothesized that APLN upregulates IL-1β expression by modulating miRNA expression in OASFs.

## RESULTS

### APLN expression is positively correlated with IL-1β expression in OA patients

**Figure 1:**
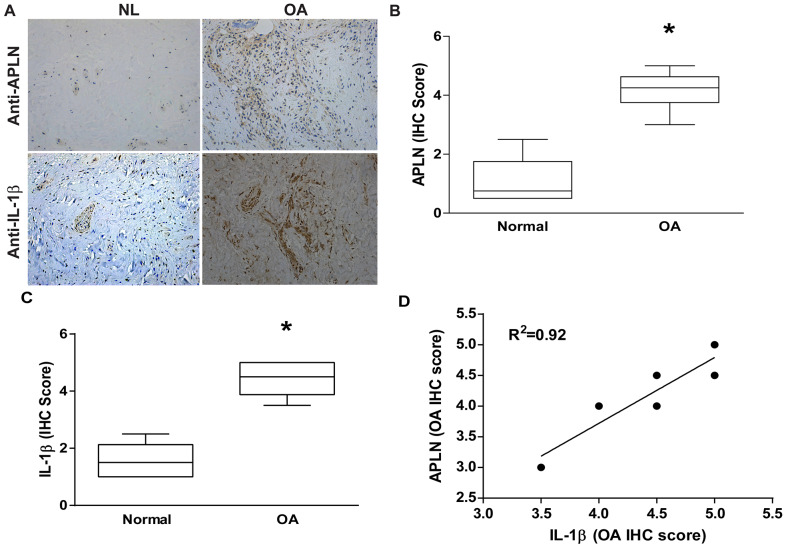
**APLN expression is positively correlated with IL-1β expression in OA patients.** (**A**) IHC staining showing increased levels of APLN and IL-1β expression in OA synovial tissue (n=8) compared to normal healthy tissue (n=5). (**B**, **C**) The IHC score of APLN and IL-1β are presented. (**D**) Correlation between levels of APLN and IL-1β expression in synovial tissues retrieved from OA patients.

To decipher crosstalk between APLN and IL-1β in the OA cohort, we used IHC staining to examine normal and OA synovial tissue samples. Levels of APLN and IL-1β expression were significantly higher in OA tissue than in normal tissue according to IHC staining (Figure 1A–1C, respectively). A positive correlation was observed between APLN and IL-1β in IHC stain (Figure 1D).

### APLN stimulates IL-1β expression in human OASFs

**Figure 2:**
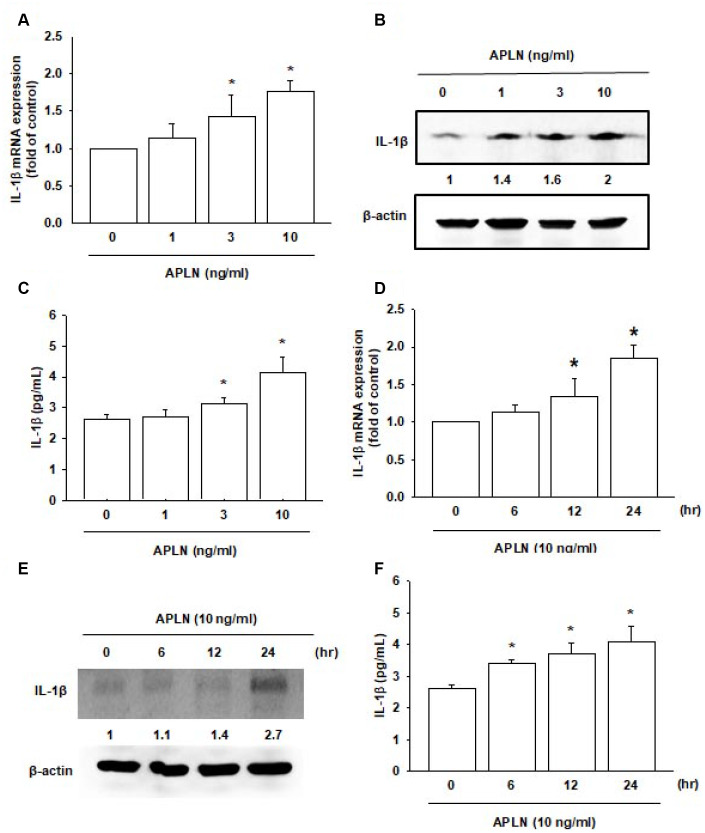
**APLN stimulates IL-1β expression in OASFs in concentration- and time-dependent manners.** (**A**) Human OASFs were incubated with 0, 1, 3, and 10 ng/mL of APLN for 24 h, and IL-1β mRNA expression levels were examined by RT-qPCR (n=4). (**B**) OASFs were incubated under various concentrations of APLN for 24 h, and IL-1β expression levels were examined by Western blot (n=3). (**C**) OASFs were cultured under various concentrations of APLN for 24 h, and excreted IL-1β were examined by ELISA assay (n=5). (**D**) OASFs were incubated with 10 ng/mL of APLN for 0, 6, 12, and 24 h. IL-1β mRNA levels were examined by RT-qPCR (n=4). (**E**) IL-1β protein synthesis levels were examined by Western blot (n=3). (**F**) Excretion of IL-1β protein levels in human OASFs was examined by ELISA (n=5). * *p*<0.05 compared with control group.

Both APLN and IL-1β are known to act as proinflammatory mediators in arthritic disease [3]. However, no detailed information exists regarding any crosstalk between them in the pathogenesis of OA nor on how such an interaction may influence the synovium-induced inflammation in OASFs. APLN (0–10 ng/mL) dose-dependently stimulated IL-1β transcription and translation (Figure 2A and 2B, respectively) and the excretion of IL-1β protein by OASFs (Figure 2C). Treatment of OASFs with APLN (10 ng/mL) for 24 hours stimulated IL-1β gene transcription and translation, as well as IL-1β protein excretion, in a time-dependent manner, as determined by RT-qPCR Western blot and ELISA assays, respectively (Figure 2D–2F). However, stimulation of OASFs with APLN did not significantly increase TNF-α expression (Supplementary Figure 1). These findings indicate that APLN enhances the downstream expression of IL-1β in human OASFs, via concentration- and time-dependent manners.

### APLN stimulates IL-1β expression via phosphoinositide 3-kinase (PI3K) and extracellular-signal-regulated kinase (ERK)

**Figure 3:**
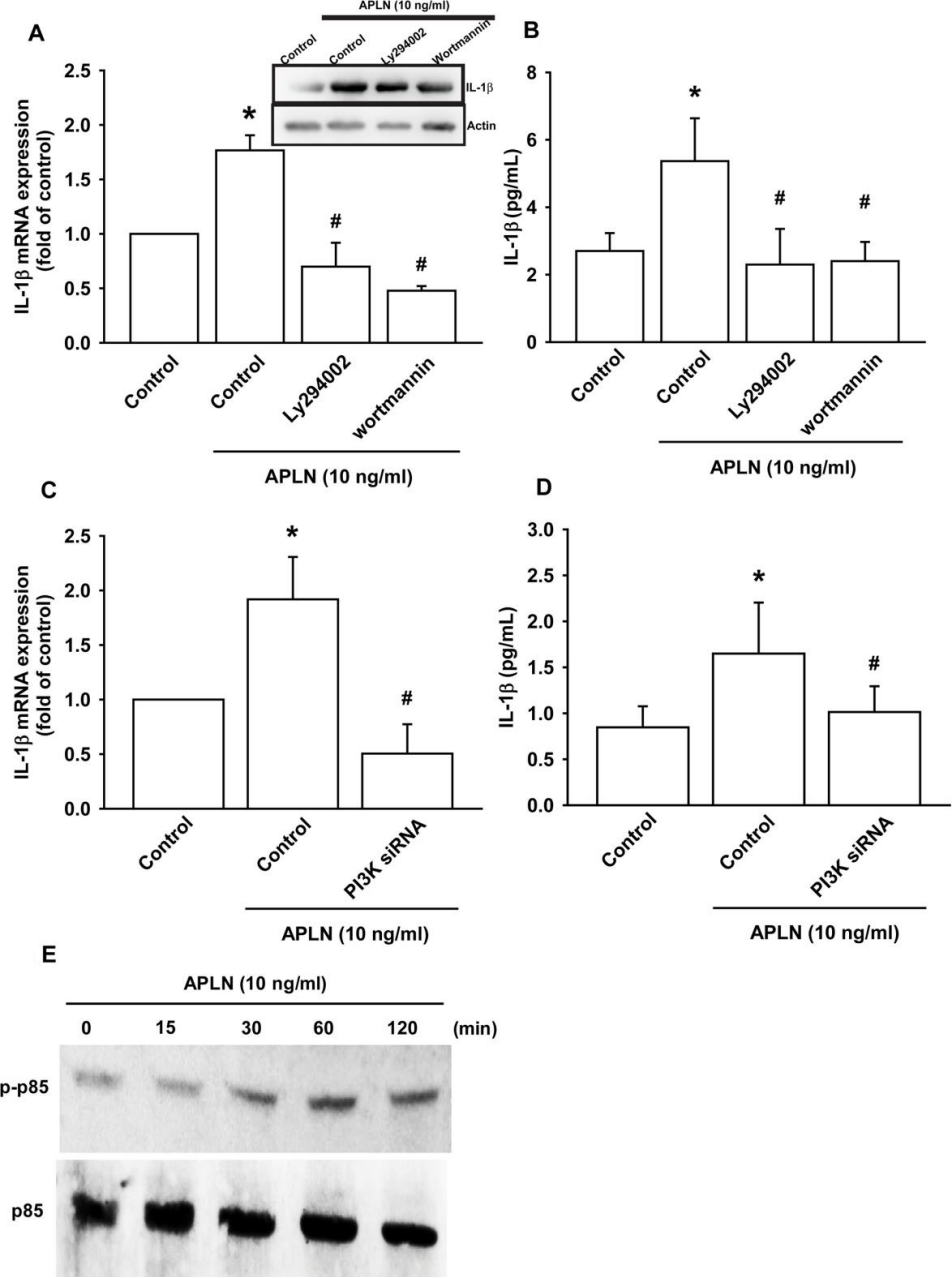
**PI3K phosphorylation is involved in APLN-induced IL-1β synthesis.** (**A**) OASFs were pretreated with PI3K inhibitors (LY294002, Wortmannin; 10 μM) for 30 min, then incubated with APLN (10 ng/mL) for 24 h. IL-1β mRNA and protein levels were examined by RT-qPCR (n=4) and Western blot (n=3) assays, respectively. (**B**) OASFs were pretreated with PI3K inhibitors (LY294002, Wortmannin; 10 μM) for 30 min, then incubated with APLN (10 ng/mL) for 24 h. Excreted IL-1 β protein levels were examined by ELISA (n=5). (**C**) OASFs were transfected with PI3K siRNA (1 μg) then incubated with APLN (10 ng/mL) for 24 h. IL-1β mRNA levels were examined by ELISA assay (n=5). (**D**) OASFs were transfected with PI3K siRNA (1 μg), then incubated with APLN (10 ng/mL) for 24 h. Excreted IL-1β protein levels were examined by ELISA assay (n=5). (**E**) OASFs were incubated with APLN for the indicated time intervals, and the extent of PI3K phosphorylation was examined by Western blot (n=3). * *p*<0.05 compared with control group; # *p*<0.05 compared with the APLN-treated group.

The PI3K enzyme is modulated by many stimuli, including APLN [21, 22]. To validate the role of PI3K in APLN-enhanced IL-1β production, we pretreated OASFs with PI3K inhibitors (LY294002, Wortmannin) or transfected them with PI3K siRNAs. RT-qPCR and Western blot assays confirmed that both PI3K inhibitors and PI3K siRNAs significantly mitigated APLN-enhanced IL-1β synthesis in OASFs (Figure 3A–3D). Moreover, Western blot demonstrated that APLN-induced stimulation of OASFs time-dependently increased the phosphorylation of p85, the PI3K regulatory subunit (Figure 3E).

**Figure 4:**
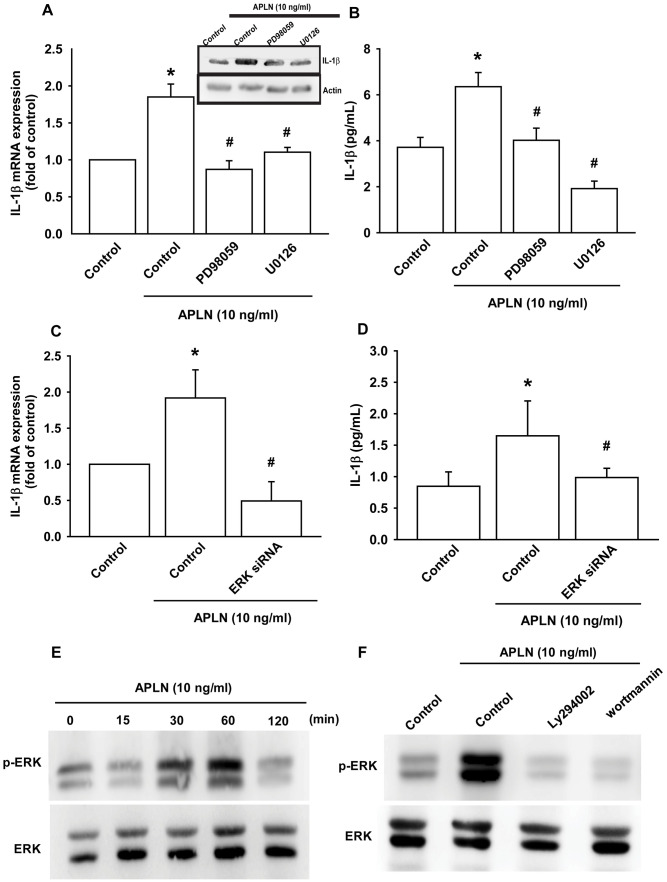
**ERK phosphorylation is involved in APLN-induced IL-1β synthesis.** (**A**) OASFs were pretreated with ERK inhibitors (PD98059, U0126; 10 μM) for 30 min, then incubated with APLN (10 ng/mL) for 24 h. IL-1β mRNA and protein levels were examined by RT-qPCR (n=4) and Western blot (n=3) assays, respectively. (**B**) OASFs were pretreated with ERK inhibitors (PD98059, U0126; 10 μM) for 30 min, then incubated with APLN (10 ng/mL) for 24 h. Excreted IL-1β protein levels were examined by ELISA (n=5). (**C**) OASFs were transfected with ERK siRNA (1 μg), then incubated with APLN (10 ng/mL) for 24 h. IL-1β mRNA levels were examined by ELISA assay (n=5). (**D**) OASFs were transfected with ERK siRNA (1 μg), then incubated with APLN (10 ng/mL) for 24 h. Excreted IL-1β protein levels were examined by ELISA assay (n=5). (**E**) OASFs were incubated with APLN (10 ng/mL) for the indicated time intervals, and the extent of ERK phosphorylation was examined by Western blot (n=3). (**F**) OASFs were pretreated with LY294002 and Wortmannin (10 μM) for 30 min, then incubated with APLN (10 ng/mL) for 24 h. The extent of ERK phosphorylation was examined by Western blot (n=3). * *p*<0.05 compared with control group; # *p*<0.05 compared with the APLN-treated group.

The protein kinase intracellular signaling molecule, extracellular signal-regulated kinase (ERK), regulates cellular proliferation and differentiation. ERK is phosphorylated by various stimuli, including growth factors, cytokines and carcinogens, and is implicated in Wogonin-mediated anti-inflammatory and protective effects in human OA chondrocytes [23]. The Ras-ERK and PI3K-mechanistic target of rapamycin (PI3K-mTOR) signaling pathways have long been considered to act as linear signaling conduits that are activated by different stimuli, but recently it has become clear that ERK and PI3K regulate each other by intersecting and co-regulating downstream functions [24]. In this study, treatment of OASFs with ERK inhibitors (PD98059, U0126) and the transfection of OASFs with ERK siRNA prior to APLN administration significantly mitigated APLN-enhanced IL-1β synthesis (Figure 4A–4D). In Western blot analysis, APLN time-dependently stimulated ERK phosphorylation (Figure 4E), which was mitigated by PI3K inhibitors (LY294002, Wortmannin) (Figure 4F). These findings suggest that APLN enhances IL-1β expression through the PI3K and ERK signaling pathways and that PI3K transmits information upstream of ERK in this cascade.

### APLN enhances IL-1β expression by inhibiting miR-144-3p synthesis

**Figure 5:**
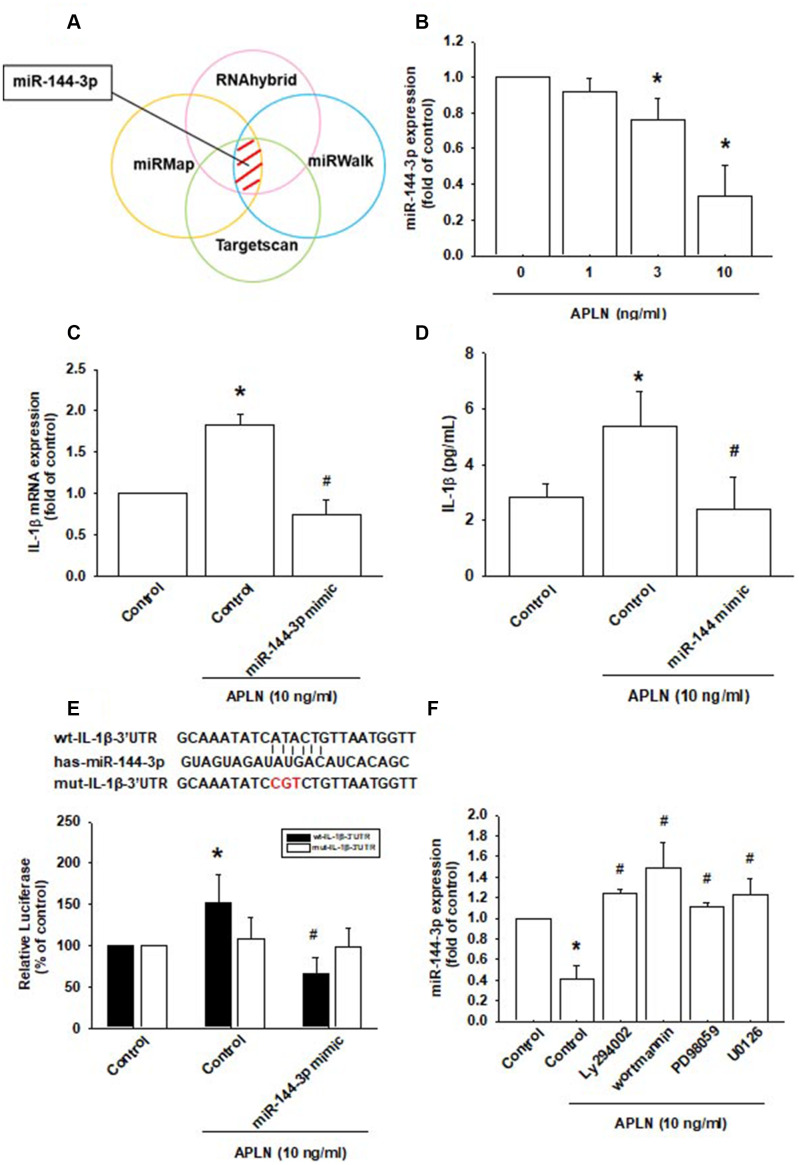
**APLN-induced suppression of miRNA-144-3p enhances IL-1β production.** (**A**) Open-source software (TargetScan, miRMap, RNAhybrid, and miRWalk) was used to identify which miRNAs could possibly interfere with IL-1β transcription. (**B**) OASFs were incubated with APLN (0, 1, 3, and 10 ng/mL). Levels of miR-144-3p expression were examined by RT-qPCR assay (n=4). (**C**, **D**) OASFs were transfected with miR-144-3p mimic and then stimulated with APLN (10 ng/mL). mRNA and excreted protein levels were examined by RT-qPCR (n=4) and ELISA assays (n=5). (**E**) OASFs were transfected with the mut-IL-1β-3′UTR plasmid with or without miRNA-144-3p mimic, then stimulated with APLN (10 ng/mL). Relative luciferase activity reflected IL-1β promoter activity (n=6). (**F**) OASFs were treated with PI3K or ERK inhibitor then incubated with APLN. miR-144-3p expression levels were examined by RT-qPCR assay (n=4). Results are expressed as the mean ± S.E.M. * *p*<0.05 compared with the control group; # *p*<0.05 compared with the APLN-treated group.

Various miRNAs demonstrate differential expression patterns between OA and normal cartilage and are involved in the pathogenesis of OA [1, 8]. However, the miRNA networks involved in OA pathogenesis are far from clear. We used open-source software (TargetScan, miRMap, RNAhybrid, and miRWalk) to identify miRNAs that could possibly interfere with IL-1β transcription (Figure 5A; Supplementary Table 1). Among the 15 candidate miRNAs that could possibly bind to the 3’UTR region of IL-1β mRNA, levels of miR-144-3p expression were significantly decreased by the greatest extent after APLN administration. To confirm these findings, we compared levels of miR-144-3p expression in OASFs treated with APLN 1–10 ng/mL. APLN concentration-dependently inhibited miR-144-3p expression (Figure 5B). To further determine whether APLN stimulates IL-1β expression by inhibiting miR-144-3p synthesis, we transfected OASFs with miR-144-3p mimic and observed reductions in APLN-enhanced IL-1β mRNA and protein secretion (Figure 5C and 5D).

We also used the luciferase reporter vector, including the wild-type 3’UTR of IL-1β mRNA (wt-IL-1β-3’UTR) and the mutated vector harboring mismatches in the predicted miR-144-3p binding site (mut-IL-1β-3’UTR), to determine whether miR-144-3p regulates transcription of the IL-1β gene (Figure 5E). miR-144-3p mimic reduced APLN-enhanced luciferase activity in the wt-IL-1β-3’UTR plasmid, but not in the mt-IL-1β-3’UTR plasmid (Figure 5E). In addition, the PI3K inhibitors (LY294002, Wortmannin) and ERK inhibitors (PD98059, U0126) significantly reversed APLN-inhibited miR-144-3p expression (Figure 5F). It appears that miR-144-3p directly suppresses IL-1β transcription by binding to the 3'UTR region of human IL-1β mRNA, and that miR-144-3p expression is negatively regulated by PI3K and ERK phosphorylation induced by upstream APLN signaling.

### Knockdown of APLN mitigates histologic features of OA

**Figure 6:**
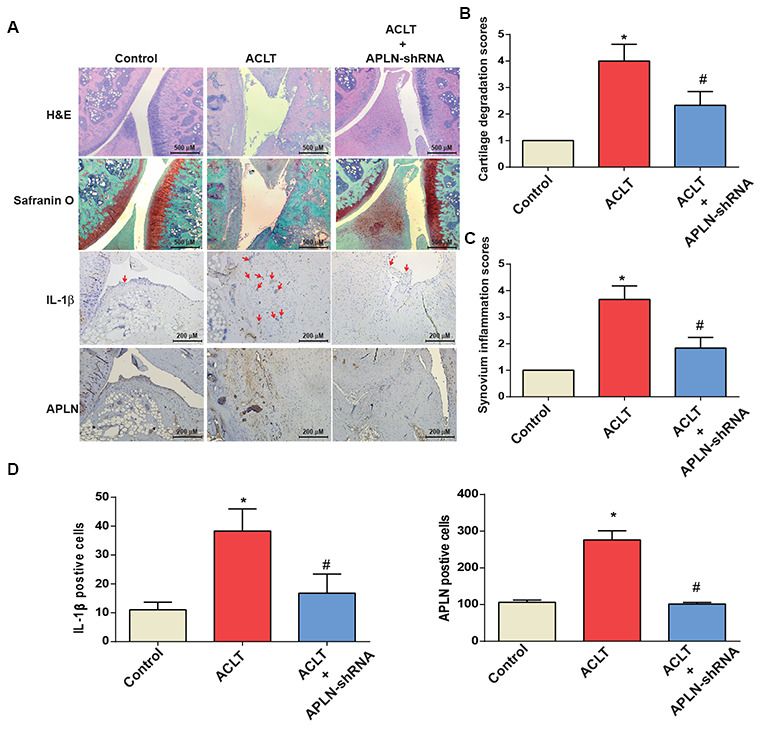
**shAPLN administration mitigates the histologic severity of OA.** (**A**) Staining of specimens with H&E, Safranin-O, IL-1β and APLN from the control knee (n=6), ACLT knee (n=6), and shAPLN-transfected ACLT knee (n=6). (**B**) Cartilage degeneration scores were calculated for articular cartilage sections stained with Safranin-O. (**C**) Synovial membrane inflammation score. Magnified area of synovium used to generate synovial inflammation score in all samples. Scoring was performed in H&E-stained slides. (**D**) IHC analysis of proportions of IL-1β-positive cells (red arrows) and APLN-positive cells in synovial lining tissues in specimens from control knees (n=6), ACLT knees (n=6), and shAPLN-transfected ACLT knees (n=6). * *p*<0.05 compared with the control group; # *p*<0.05 compared with the control shRNA-transfected ACLT group.

To validate the *in vivo* role of APLN, we investigated the effects of shRNA-mediated APLN knockdown in mitigating disease severity in the ACLT OA model. Compared with control samples, ACLT samples transfected with control shRNA exhibited significantly lower cartilage thickness in Safranin-O and H&E staining (Figure 6A), and a significantly higher proportion of IL-1β- and APLN-positive synovial cells in IHC analysis (Figure 6B–6D). ACLT-induced histologic changes were reversed by downregulating APLN expression.

## DISCUSSION

APLN has a crucial role in the pathogenesis of arthritic diseases, including rheumatoid arthritis and OA [3]. Early *in vitro* investigations indicated that APLN significantly increases MMP-1, -3 and -9 transcript levels, and IL-1β protein synthesis in chondrocytes [14]. In that same study, rats administered intra-articular injections of APLN (1 nM) experienced significant increases in gene expression levels of MMP-1, -3 and -9, A disintegrin and metalloproteinase with thrombospondin motifs 4 (ADAMTS-4) and -5, and IL-1β, while the gene expression of collagen II was significantly decreased by APLN; moreover, protein syntheses of MMP-3, -9, and IL-1β were significantly increased and synthesis of collage II decreased by APLN, compared with control samples. APLN also lowered cartilage proteoglycan production in articular cartilage [14]. The impact of APLN on OASFs has not been previously clarified. Here we reported that APLN levels were higher in OA tissue than in normal tissue. We did not examine APLN expression in other articular tissues such as cartilage and muscle, blood vessels, or inflammatory cells. The autocrine- and paracrine-driven aspects of APLN-induced effects in the articular microenvironment deserve further study. In this study, we found that APLN stimulates IL-1β expression in OASFs by stimulating PI3K and ERK phosphorylation and suppressing the downstream expression of miR-144-3p. These results add to the existing literature on OA pathogenesis.

Many miRNAs are involved in OA pathogenesis [25, 26]. We used open-source software (TargetScan, miRMap, RNAhybrid, and miRWalk) to predict which miRNAs potentially interfere with APLN transcription. Among all candidate miRNAs, miR-144-3p was suppressed to the greatest extent by APLN. The analyses also showed that transfection of OASFs with miR-144-3p mimic mitigates APLN-stimulated IL-1β expression. These findings underscore the importance of miR-144-3p in the process of APLN-stimulated IL-1β expression.

The APJ receptor is a major receptor of APLN. The APLN/APJ system is a critical regulator of various physiological functions, such as glycometabolism, liver disease and macrophage activation [27–29]. This study did not examine APJ expression in synovial tissues. Further investigation needs to determine whether similar interactions are involved in APLN-induced promotion of IL-1β expression in OA synovial fibroblasts. The roles of PI3K and ERK in OA pathogenesis have been explored in previous work. The PI3K/Akt pathway is involved in both the degradation of extracellular matrix and chondrocyte death [30]. Inhibition of the PI3K/AKT/mTOR signaling pathway promotes autophagy of articular chondrocytes and attenuates inflammatory responses in rats with OA [31]. PI3K plays a pivotal role in allicin-suppressed IL-1β expression in chondrocytes [32]. In a dog model of surgically-induced OA, activation of ERK1/2, JNK and p38 was higher in OA tissue compared with normal tissue [33]. ERK is involved in the pathologic interaction between OA subchondral osteoblasts and articular chondrocytes as well as the hypertrophic differentiation of articular chondrocytes [34]. In this study, we show that APLN stimulates IL-1β expression via PI3K and ERK phosphorylation. Our data emphasize the importance of PI3K and ERK in OA pathogenesis. Transcriptional and post-transcriptional modulations affect miRNA activation and inhibition [35]. In this study, treatment of OASFs with PI3K and ERK inhibitors antagonized APLN-inhibited miR-144-3p expression, indicating that APLN suppresses the expression of miR-144-3p via PI3K/ERK signaling. Whether PI3K/ERK modulates miR-144-3p expression via post-transcriptional regulation warrants further examination.

**Figure 7:**
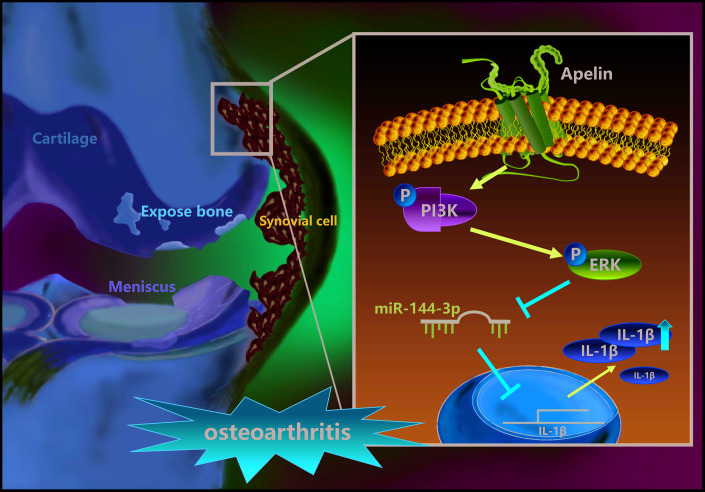
**Schematic diagram summarizes the mechanism whereby APLN promotes IL-1β production in OASFs.** APLN induces inflammatory IL-1β production in OASFs by downregulating miR-144-3p through the PI3K and ERK signaling pathways.

In summary, our study shows that APLN treatment of OASFs triggers PI3K and ERK phosphorylation and contributes to a decline in miR-144-3p expression (Figure 7). These results improve our understanding about the role of OASFs in the pathogenesis of obesity and OA and may lead to the design of more effective therapy for OA patients.

## MATERIALS AND METHODS

Antibodies against IL-1β, PI3K, and ERK were all bought from Santa Cruz (Santa Cruz, CA, USA). Antibodies against p-PI3K and p-ERK were purchased from Cell Signaling (Cell Signaling, UK). ON-TARGETplus siRNAs for IL-1β, PI3K, ERK and the control siRNA were purchased from Dharmacon (Lafayette, CO, USA). PI3K inhibitors (LY294002, Wortmannin) and ERK inhibitors (PD98059, U0126) were supplied by Calbiochem (San Diego, CA, USA). Cell culture supplements were purchased from Invitrogen (Carlsbad, CA, USA). A Dual-Luciferase^®^ Reporter Assay System was bought from Promega (Madison, WI, USA). The qPCR primers and probes, as well as the Taqman^®^ one-step PCR Master Mix, were supplied by Applied Biosystems (Foster City, CA, USA). All other chemicals not mentioned above were supplied by Sigma-Aldrich (St. Louis, MO, USA).

### Cell culture

Synovial tissue from the suprapatellar pouch of the knee was obtained from 20 patients diagnosed with Ahlbäck stage IV OA. Synovial fibroblasts were cultured in DMEM medium supplemented with 10% fetal bovine serum (FBS), 50 units/mL penicillin and 50 μg/mL streptomycin, as previously described [1, 36].

### Clinical samples

Synovial tissue samples were obtained from patients with OA undergoing knee replacement surgery and those undergoing arthroscopy after trauma/joint derangement (these were used as healthy controls) at China Medical University Hospital. The study protocol was approved by the Institutional Review Board (IRB) of China Medical University Hospital and all methods were performed in accordance with the IRB’s guidelines and regulations. Informed written consent was obtained from all patients.

### Real-time quantitative PCR analysis of mRNA and miRNA

Total RNA was extracted from human synovial fibroblasts by TRIzol; reverse transcription used 1 μg of total RNA transcribed into cDNA by oligo (dT) primers. Real-time quantitative PCR (RT-qPCR) used the Taqman^®^ One-Step RT-PCR Master Mix. All RT-qPCR assays were performed using the StepOnePlus sequence detection system (Applied Biosystems) [37, 38].

### Western blot analysis

Cell lysate was separated by SDS-PAGE electrophoresis then transferred to polyvinylidene difluoride (PVDF) membranes, following the method described in our previous work [39]. After blocking the blots with 4% bovine serum albumin, the blots were treated with primary antibody and then secondary antibody. Enhanced chemiluminescent imaging of the blots was visualized with the UVP Biospectrum system (UVP, Upland, CA, USA) [40–42].

### Plasmid construction and luciferase assays

Wild-type and mutant IL-1β 3’-UTRs were generated on the miR-144-3p target recognition sites (seed sequences). The wild-type 3’-UTRs of IL-1β were cloned into the pmirGLO-luciferase reporter vector using Nhe1 and Xho1 restriction sites. The primer sequences used were defined as the IL-1β forward primer (CGGCTAGCAGAAACCACGGCCACATTT) and the reverse primer (GGCTCGAGTTCAGTGAAGTTTATTTCAGAACCA). All constructs were sequenced to verify that they contained the 3’-UTR inserts. The mutant 3’UTR region of IL-1β mRNA (mut-IL-1β 3’-UTR) was purchased from Invitrogen. Luciferase activity was assayed using the method described in our previous publications [1, 8, 43].

### Lentiviral production

Recombinant lentiviruses were produced by transient cotransfection of 293T cells with short hairpin (sh)RNA-expressing plasmid (TRCN0000004877) with the packaging plasmid pCMVDR8.91 and the VSV-G envelope glycoprotein expression plasmid pMD.G. All were obtained from the National RNAi Core Facility at the Academia Sinica in Taiwan. After 48 hours, lentiviral particles carrying shAPLN (Lenti-shAPLN) were isolated from the supernatant of 293T cells. A plaque assay using serial dilutions was performed in OASFs and determined the viral titer of Lenti-shAPLN to be ~7.1 x 10^6^ plaque-forming units (PFU)/mL.

### Experimental OA model

Sprague-Dawley (SD) rats (8 weeks of age, weighing 300–350 g) were purchased from the National Laboratory Animal Center in Taiwan and maintained under conditions complying with the Guidelines of the Animal Care Committee of China Medical University, Taichung, Taiwan, as described in our previous work [44]. We followed the protocol established by Wang et al. for our ACLT model to induce OA in rats [45]. In brief, the right knee was prepared in a surgically sterile fashion. Using a medial parapatellar mini-arthrotomy approach, the ACL fibers were transected with a scalpel and the entire medial meniscus was excised. After surgery, the joint surface was washed with sterile saline solution and the capsule and skin were sutured. The rats received prophylactic antibiotic with ampicillin 50 mg/kg body weight for 5 days post-surgery. After surgery (day 0), the rats were divided into 3 groups (n=8 per group), including a control group, an ACLT group, and an shAPLN-transfected ACLT group. For 6 weeks, the shAPLN-transfected ACLT group were given weekly intra-articular injections of ~7.1 x 10^6^ PFU of APLN-shRNA. All rats were allowed to move freely in plastic cages until their necropsies at 6 weeks post-surgery.

### Histological analysis

Immunohistochemistry (IHC) staining was performed on serial sections of the mice knee joints. After fixing the knee joints in 1% formaldehyde, the specimens were decalcified in 10% EDTA and dehydrated in ethanol/xylene, following previous work [46]. All sections were stained with primary anti-IL-1β (1:200) (Santa Cruz Biotechnology). Biotin conjugated goat anti-rabbit immunoglobulin G (IgG) was used as the secondary antibody and 3,3ʹ-diaminobenzidine tetrahydrochloride as the substrate for color development. Some specimens were also stained with Safranin-O/Fast-green or hematoxylin and eosin (H&E) [44, 46].

The cartilage degeneration score evaluates overall cartilage pathology and includes the important parameters of collagen matrix fibrillation/loss and chondrocyte death/loss, with chondrocyte loss being the primary determinant of the score (Grade 0 = no changes; Grade 1 = minimal degeneration, with 5–10% of the total projected cartilage area affected by matrix or chondrocyte loss; Grade 2 = greater than mild degeneration, with 11–25% of the area affected; Grade 3 = greater than moderate degeneration, with 26–50% of the area affected; Grade 4 = marked degeneration, with 51–75% of the area affected; Grade 5 = more severe degeneration, with over 75% of the area affected).

Synovial membrane inflammation was scored as follows: Grade 0 = no changes (1–2 layers of synovial lining cells); Grade 1 = an increased number of lining cell layers (≥3–4 layers) or slight proliferation of subsynovial tissue; Grade 2 = an increased number of lining cell layers (≤3–4 layers) and/or proliferation of subsynovial tissue; Grade 3 = an increased number of lining cell layers (>4 layers) and/or proliferation of subsynovial tissue and infiltration of few inflammatory cells; Grade 4 = an increased number of lining cell layers (>4 layers) and/or proliferation of subsynovial tissue with infiltration of a large number of inflammatory cells.

### Statistics

All values are given as the mean ± standard error of the mean (S.E.M.). The Student’s *t*-test assessed between-group differences. A *p* value of <0.05 was considered to be statistically significant.

## Supplementary Material

Supplementary Figure 1

Supplementary Table 1
