# Supplementary material for: Apelin enhances IL-1β expression in human synovial fibroblasts by inhibiting miR-144-3p through the PI3K and ERK pathways
Source: Aging (Albany NY). 2020 May 18;12(10):9224–39. doi: 10.18632/aging.103195 (PMC7288923; doi:10.18632/aging.103195)
Supplement: Supplementary Figure 1 [file aging-12-103195-s001..pdf]

SUPPLEMENTARY FIGURE

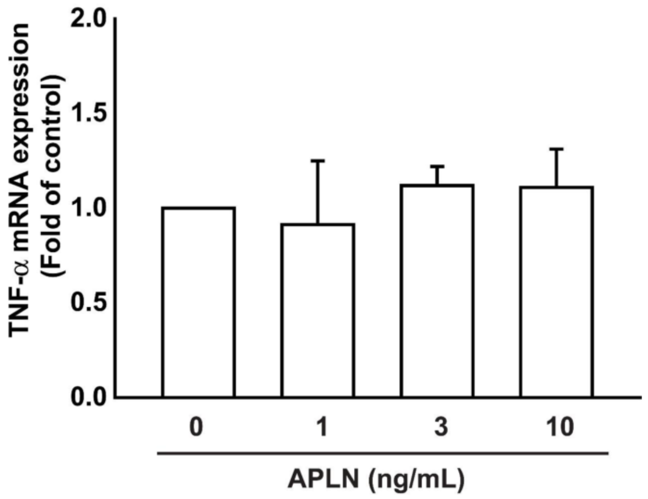

Supplementary Figure 1. Stimulation of OASFs with APLN did not significantly increase TNF-α expression. After incubating human OASFs with 0, 1, 3, or 10 ng/mL of APLN for 24 h, TNF-α mRNA expression levels were examined using RT-qPCR analysis (n=4).
