# Supplementary material for: Apelin enhances IL-1β expression in human synovial fibroblasts by inhibiting miR-144-3p through the PI3K and ERK pathways
Source: Aging (Albany NY). 2020 May 18;12(10):9224–39. doi: 10.18632/aging.103195 (PMC7288923; doi:10.18632/aging.103195)
Supplement: Supplementary Table 1 [file aging-12-103195-s002..pdf]

SUPPLEMENTARY TABLE

Supplementary Table 1. Open-source software programs (TargetScan, miRMap, RNAhybrid, and miRWalk) predicted miRNAs that may interfere with IL-1β transcription.

miRNAs identified from open-source softwares
miR-21-5p, miR-27b-5p, miR-34b-5p, miR-124,<br>miR-144-5p, miR-149-5p, miR-181a-5p, miR-181c-5p,<br>miR-185-5p, miR-204-5p, miR-211-5p, miR-219a-5p,<br>miR-296-5p, miR-340-5p, miR-345-5p

The predicted miRNA results were made using miRWalk2.0 (http://zmf.umm.uni-heidelberg.de/apps/zmf/mirwalk2/). The detailed protocol is as follows:

1. Click on "Predicted Target Module" and select "Gene-miRNA Targets".
2. In Step 1, select "NCBI" and enter the gene ID (3553; IL-1β).
3. In Step 3, in the other databases section, select all databases and choose the command "OR" against each database.
4. In Step 4, select "SEARCH".
5. On the new page that appears, under the header "Putative miRNA binding sites predicted by chosen algorithms within mRNA selected regions", select "3UTR".
6. This reveals many predicted miRNAs that bind to IL-1β 3'-UTR, ranging in scores from high to low. Fifteen high-scoring candidate miRNAs were used in this study.
